# Characterization of SARS-CoV-2 East Java isolate, Indonesia

**DOI:** 10.12688/f1000research.53137.1

**Published:** 2021-06-16

**Authors:** Fedik Abdul Rantam, Cita Rosita Sigit Prakoeswa, Damayanti Tinduh, Jusak Nugraha, Helen Susilowati, Andi Yasmin Wijaya, Ni Nyoman Tri Puspaningsih, Dwiyanti Puspitasari, Dominicus Husada, Neneng Dewi Kurniati, Aryati Aryati

**Affiliations:** 1 Research Center for Vaccine Technology and Development, Institute of Tropical Disease, Airlangga University, Surabaya, East Java, 60132, Indonesia; 2 Virology and Immunology Laboratory, Department of Microbiology, Faculty of Veterinary Medicine, Airlangga University, Surabaya, East Java, 60132, Indonesia; 3 Professioal Education and Research, Dr. Soetomo General Academic Hospital, Faculty of Medicine, Airlangga University, Surabaya, East Java, 60132, Indonesia; 4 Research and Development Board, Dr. Soetomo General Hospital, Surabaya, East Java, 60132, Indonesia; 5 Clinical Pathology Department, Dr. Soetomo General Hospital, Faculty of Medicine, Airlangga University, Surabaya, East Java, 60132, Indonesia; 6 Faculty of Medicine, Airlangga University, Surabaya, East Java, 60132, Indonesia; 7 Bioresource Engineering Group in Research Center for Bio-Molecule Engineering (BIOME), Airlangga University, Surabaya, East Java, 60132, Indonesia; 8 Pediatrics Department, Dr. Soetomo General Hospital, Surabaya, East Java, 60132, Indonesia; 9 Clinical Microbiology Department, Dr. Soetomo General Hospital, Surabaya, East Java, 60132, Indonesia

**Keywords:** growth properties, COVID-19, SARS-CoV-2, virus isolation, infectious disease

## Abstract

**Background:** Incidents of SARS-CoV-2 in East Java increased steadily, and it became the second epicenter in Indonesia. The COVID-19 pandemic caused a dire multisectoral crisis all around the world. This study investigates and characterizes local isolates from East Java, Indonesia.

**Methods:** There were 54 patients suspected with SARS-COV-2 infection and 27 patients were COVID-19 positive. Virus isolates were obtained from COVID-19 inpatients’ nasopharyngeal swabs at the Dr Soetomo Teaching Hospital, Surabaya. There were only three isolates (#6, #11, #35) with good growth characteristics. Serial blind passage and cytopathic effect observation in the Vero E6 cell line were performed for virus isolation. Confirmation of the SARS-CoV-2 infection was proven by means of reverse transcriptase-polymerase chain reactions using SARS-CoV-2 specific primers, scanning electron microscopy, and scanning transmission electron microscopy examination. Whole genome sequencing was performed using ARTIC protocol. Furthermore, SARS-CoV-2 characterization was identified through a western blot using rabbit serum immunized with inactive SARS-CoV-2 vaccine and human natural COVID-19 infection serum.

**Results:** Spike gene analysis of three samples (#6, #11, #35) found that the D614G mutation was detected in all isolates, although one isolate exhibited the D215Y and E484D mutation. Based on whole genome analysis, those three isolates were included in clade 20A, and two isolates were included in lineage B.1.6 with one isolate belongs to lineage B.1.4.7.

**Conclusion:** Based on molecular characterization and immunogenicity of SARS-CoV-2 East Java, Indonesia showed high titer and it has mutation in some regions.

## Introduction

Coronaviruses are an enveloped positive single-stranded ribonucleic acid (RNA) virus that can infect several species via zoonotic transmission. ^1, 2^ The coronavirus viral particle is a heterogenous, spherical, crown shaped viral particle with a diameter ranging from 80–160 nm. ^2^ Severe acute respiratory syndrome coronavirus 2 (SARS-CoV-2) possesses several main structural proteins including envelope, membrane, nucleoprotein, and spike protein. ^3^ Spike protein facilitates the SARS-CoV-2 infection interacting with the human angiotensin-converting enzyme-2 (ACE-2) protein, which acts as a receptor, and it is expressed in various tissues in the body. ^4^ This abundant presence of a virus receptor could be responsible for its rapid spread of infection. In addition, spike protein as the main protein of interest such as envelope, membrane, and nucleoprotein could also have an immunogenic capability. ^3^ Worldwide genomic surveillance has proven that the spike protein exhibits a tendency to have multiple sites of mutation.

Several major mutations of concern in SARS-CoV-2 target the spike protein such as in the UK (lineage B.1.1.7), South Africa (B.1.351), and Brazil (lineage P.1), and caused concern as these mutations were responsible for enhancing SARS-CoV-2 infection morbidity and resistance of serum neutralization. ^5– 7^ However, these mutations were not proven to correlate with COVID-19 severity and mortality.

The SARS-CoV-2 infection caused the Coronavirus Disease 2019 (COVID-19) that was first reported in Wuhan, China, in December, 2019 and escalated quickly until it was declared a global pandemic by the World Health Organization (WHO) in March 2020. ^8, 9^ SARS-CoV-2 is spread through droplets and aerosol-mediated infection that relate to COVID-19 primary symptoms of respiratory complaints that vary from mild symptoms to dire acute respiratory distress syndrome (ARDS). ^10^ However, in a recent development, it has been reported that the SARS-CoV-2 infection can spread from the respiratory tract port of entry to the whole human body, and can cause various clinical manifestations, from gastrointestinal symptoms (diarrhea, nausea, and vomiting), neurological symptoms (anosmia and decrease of taste senses), ophthalmological symptoms (conjunctivitis), nephrological symptoms (acute kidney injury), hypercoagulability state, and systemic viral sepsis. ^11, 12^

Indonesia, one of the tropical countries in Southeast Asia, began to detect and report its patient zero on March 2, 2020, in Jakarta. After the first two confirmed cases were declared positive for infection of SARS-CoV-2, the infection began to spread across the Indonesian archipelago. ^13, 14^ In May, 2020, the incidence of SARS-CoV-2 increased steadily in Surabaya, making it the second epicenter in Indonesia after Jakarta. ^15^

Indonesia is the fourth most populated country in the world to be affected by the COVID-19 pandemic, and various areas of national concern have been impacted, including economics, politics, and human welfare. ^16, 17^ Researchers around the world are now racing against SARS-CoV-2, aiming to control its impact with a holistic approach. ^18^ Development and evaluation of COVID-19 diagnostic tools, clinical management, and vaccine candidates are known to be global priorities, especially the protection of healthcare providers as frontline workers in pandemic management and mitigation, as they are more exposed to COVID-19. ^19, 20^ As SARS-CoV-2 spread around the globe, it was revealed that, based on the reported SARS-CoV-2 whole genome database, SARS-CoV-2 had already undergone several genetic mutations from its ancestor, generating evidence for distinct lineages by the summer of 2020. ^8^ Therefore, it has been suggested that isolation and characterization of SARS-CoV-2 strains from various places is required to ensure a compatible tailor-made SARS-CoV-2 management plan for specific conditions. ^20, 21^ SARS-CoV-2 genomes that have been reported in Indonesia consist of predominantly lineage B.1 based on the GISAID SARS-CoV-2 genome database and they are divergent from the Wuhan isolate. ^21^

It is reported that at the population level, there is relatively insufficient herd immunity to drive significant mutation, and there are several spike mutations that increase virus transmission without changes in clinical significance such as D614G. Moreover, an immune system exposed to a variant of SARS-CoV-2 can be active also on other variants, given the generation of polyclonal antibodies for multiple epitopes. ^22, 23^

This is the first report characterizing the SARS-CoV-2 East Java, Indonesia local isolates. This study collected SARS-CoV-2 material from three virus isolates and infected Vero E6 cells. Furthermore, this study aims to investigate the characterization of SARS-CoV-2 East Java, Indonesia local isolates.

## Methods

### Subject characteristics

Subject data was retrieved from Dr Soetomo General Hospital medical records. The first subject, (#35 subject), was a local transmission case of a 49-year-old man with a normal body mass index (22.9 kg/m ^2^), who was discharged on the 15th day of hospital care. The second subject, (#6 subject), was a local transmission case of a 38-year-old woman with an overweight body mass index (25.2 kg/m ^2^) and who rapidly tested positive for IgG with IgM antibodies. Subject #6 was admitted to inpatient care and deteriorated until being declared brain dead after six days of hospitalization due to respiratory failure. The third subject, (#11 subject), was a local transmission case of a 64-year-old woman with a normal body mass index (19.5 kg/m ^2^) and a history of breast cancer, and was in remission. Subject #11 was discharged after 26 days of hospitalization.

### Specimen collection

There were 54 patients suspected with SARS-COV-2 infection and of these 27 patients were diagnosed COVID-19 positive during March to June 2020. A SARS-CoV-2 sample source was retrieved from COVID-19 confirmed inpatients in the Dr Soetomo General Hospital and three isolates originating from three individual subjects from Surabaya, Indonesia were obtained. Each subject was asked for informed consent. A nasopharyngeal swab was performed to obtain a SARS-CoV-2 sample using a sterile cotton swab. The sample cotton swab was then submerged in virus transport medium (VTM) containing a sterile filtered solution of Minimum Essential Media (MEM) (Gibco, USA), 1x penicillin-streptomycin (Gibco, USA), and 1x amphotericin B (Gibco, USA) as reported before. ^24^ The sample was then immediately transferred, in a 4°C cool box containing an icepack, to the Research Center for Vaccine Technology and Development, Institute of Tropical Diseases Laboratory, Biosafety Level-3 (BSL-3) facility in Universitas Airlangga, Surabaya, East Java, Indonesia. ^25^

### SARS-CoV-2 isolation

**Figure 1.**
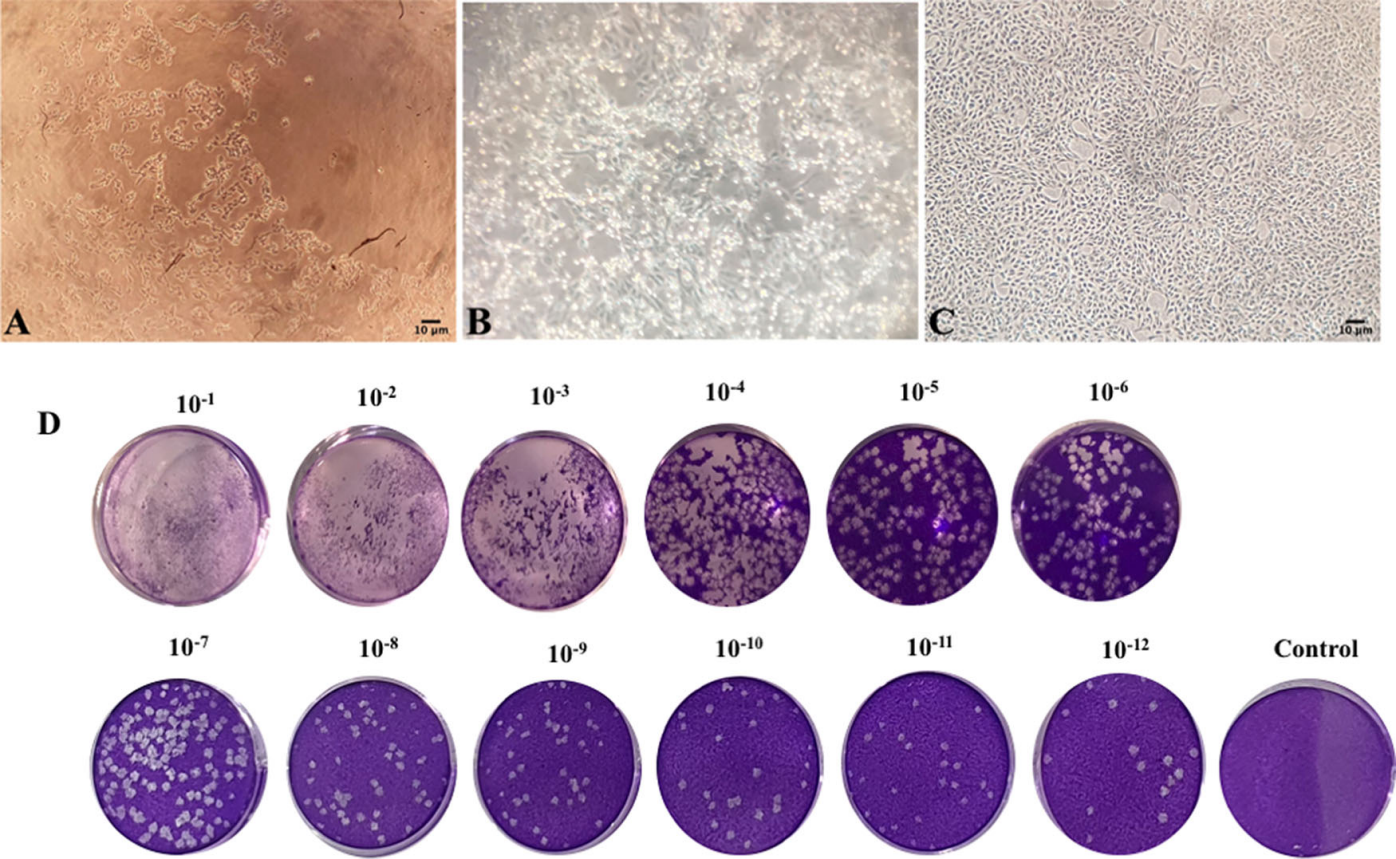
(A) Cytopathic effect of infected Vero E6 cell in third blind passage on the eighth day post passage (40× magnification); (B) Cytopathic effect of infected Vero E6 cell in 15th blind passage on second day post passage (40× magnification); (C) Uninfected Vero E6 cell in 40× magnification; (D) Plaque-Forming Unit (PFU) evaluation (Nikon TMS inverted microscope, Japan).

There were only three samples from subjects #6, #11, #35 with good growth characteristics that provided isolates. Serial blind passage and cytopathic effect observation in the Vero E6 cell line were performed for virus isolation. The virus isolation that was performed in the Vero E6 cell line (ATCC, USA) was seeded on a T25 flask (Corning, USA) with a 2 × 10 ^6^ cell count and cultured with a MEM (Gibco, USA), supplemented with 10% Fetal Bovine Serum (FBS) (Gibco, USA) until it reached 80% confluency (37°C and 5% CO _2_), ( Figure 1), by introducing a 1 ml sample of VTM to the Vero E6 cell line culture for a one-hour incubation. After incubation, 4 ml of fresh MEM (Gibco, USA), supplemented with 10% FBS (Gibco, USA) were added. The cell was labeled as inoculation culture and observed daily through a phase contrast inverted microscope TMS (Nikon, Japan). After three days of inoculation, blind passage was performed to a fresh monolayer Vero E6 cell culture by introducing a 1 ml supernatant medium from infected cells with a one-hour incubation before adding 4 ml of fresh MEM supplemented with 10% FBS medium. Viral growth was analyzed using cytopathogenic effect (CPE) and plaque forming unit (PFU) assay. TCID50 evaluation of #35 isolate through plaque forming assay and Reed and Muench method was as follows: log10 50% end point dilution = log10 of dilution showing a mortality next above 50% - (difference of logarithms × logarithm of dilution factor). Generally, the following formula is used to calculate “difference of logarithms” (difference of logarithms is also known as “proportionate distance” or “interpolated value”): Difference of logarithms = [(mortality at dilution next above 50%)-50%] / [(mortality next above 50%) - (mortality next below 50%)]. ^26^

### SARS-CoV-2 morphology

The entire electron microscopy sample preparation was performed in a biosafety class II, type A2 biosafety cabinet (Nuaire, USA). SARS-CoV-2 morphology was assessed using a scanning electron microscope (SEM) and a scanning transmission electron microscope (STEM). Sample preparation for SEM was performed by collecting and filtering 1 ml of UV-inactivated infected medium from the virus culture during the occurrence of cytopathic effect through a 1 μm pore syringe nitrocellulose filter (Merck, USA) to trap the virus. Fixation, Periodic Acid-Schiff (PAS), was performed by filtering 2 ml of 2% glutaraldehyde (Serva, USA) through the virus-entrapped syringe filter and incubating at 4°C for four hours. The fixated syringe filter then went through a dehydration process with cold ethanol (Merck, USA), at a gradient of 50%, 70%, 85%, 95% ethanol, and absolute ethanol with 15 minutes of incubation for each sequential dehydration process. Glutaraldehyde fixation and 50–75% ethanol dehydration sequences were performed using a 4°C refrigerator in an airtight container. The 85% and absolute ethanol dehydration processes were carried out at room temperature. The nitrocellulose membrane filter was then extracted from the syringe filter using pliers and air-dried in a biosafety cabinet overnight. A FEI Quanta 650 FEG (FEI, USA) electron microscope in low vacuum SEM mode (80 Pa; 10 kV) at 80,000× magnification and Modular Automated Processing System (MAPS) software version 3.14.11 (ThermoFisher Scientific, Waltham, Massachusetts, United States) was used for sample observation.

STEM sample preparation was performed by collecting and pipetting 50μl UV-inactivated infected mediums from virus culture during the occurrence of cytopathic effect to parafilm. Immobilization of a SARS-CoV-2 viral particle was conducted on a formvar (SPI-chem, USA) coated copper electron microscope grid (EMS, USA). Formvar grid coating was performed according to UK standards for microbiology investigation protocols. ^27^ The formvar-coated grid was pushed to an infected medium droplet on parafilm and incubated for 20 minutes. Excess infected medium was absorbed by filter paper, air-dried, and fixated with 2% glutaraldehyde in an airtight container (4°C, four hours). Excess glutaraldehyde was absorbed with filter paper and the virus-seeded grid was air-dried in a biosafety cabinet overnight. Negative staining was performed by utilizing 2% phosphotungstic acid (EMS, USA) with a one-minute incubation before observation. STEM sample observation was conducted using a FEI Quanta 650 FEG (FEI, USA) electron microscope through the STEM mode (1.5°C, 676 psi, 30kV) with MAPS software. ^28^

### Reverse transcription-polymerase chain reaction (RT-PCR) and gel electrophoresis

Identification of SARS-CoV-2 virus culture was performed by total RNA extraction utilizing Trizol reagent (Thermo Fisher, USA) according to the manufacturer’s protocol. A Qubit™ RNA BR Assay Kit (Thermo Fisher Scientific, USA) with a Qubit™ fluorometer was used according to the kit manual. ^29^ RT-PCR was performed from extracted RNA samples utilizing the Goscript ^®^ RT-PCR system (Promega, USA) with SARS-CoV-2 receptor-binding domain spike gene primers adopted from previous studies (forward: 5′-CCACAGACACTTGAGATTC-3′ and reverse: 5′-GCAACTGAATTTTCTGCACCA-3′) and according to supplied protocol by Lau *et al*. ^30^

Conventional PCR was performed from RT-PCR cDNA using the previously described primer with GoTaq ^®^ green master mix (Promega, USA) in a Thermal Cycler XP machine (Bioer Technology, China). Conventional PCR conditions were conducted through initial denaturation (95°C, five minutes), amplification (45 cycles of ten seconds, 95°C), denaturation (ten seconds, 62°C) annealing, (ten seconds, 72°C) elongation, final elongation (72°C, five minutes), and PCR reaction termination (4°C, 30 minutes). PCR products were detected by performing agarose gel electrophoresis in 2% agarose gel (INTRON Biotechnology, South Korea) in 0.5x Tris-Boric-EDTA (TBE) Buffer (BIOWORLD, USA) with 110 volts for 60 minutes, the DNA band was stained with ethidium bromide (TCI, Japan), and visualized through the Gel Doc XR+ gel documentation system (Bio-Rad, USA). The PCR product length was confirmed with Image Lab software for PC version 6.1 (SOFT-LIT-170-9690-ILSPC-V-6-1, Bio-Rad Laboratories Inc, Hercules, California, US, https://www.bio-rad.com/en-id/product/image-lab-software?ID=KRE6P5E8Z) in alignment with a Thermal Cycler XP machine (Roche, USA). ^31, 32^

### Whole genome sequencing

Whole genome sequencing protocol was performed by targeted sequencing after gene amplification using ARTIC V3 primer sets RT-PCR before proceeding to PCR clean up, and processed with Nanopore sequencing kits in the GRIDION platform, according to provided protocols supplied with the kits. ^33^ Whole-genome sequencing was performed by Genetika Science Indonesia Ltd through EPI2ME Labs software version 21.05 ( https://labs.epi2me.io/wfindex) and trimming through Medaka software version 1.3.4 ( https://nanoporetech.github.io/medaka/). Gene analysis was performed by RAMPART software version 1.7.1 ( https://artic.network/ncov-2019/ncov2019-using-rampart.html) for mutation analysis and identification of the spike gene and the Wuhan Hu-1 as the reference sequence. ^34^

### Immunocytochemistry

Staining of Vero E6 SARS-CoV-2 infected cells were performed with immunized rabbit antibody serum, and visualization with diaminobenzidine (DAB) was performed with horseradish peroxidase (HRP) conjugated anti-rabbit IgG. Unspecific binding blocking was performed with 0.5% bovine serum albumin. The positive infected cells that were stained brown were observed by an inverted light microscope (Nikon TMS, Japan). ^35^

### Western blot analysis

Viral protein was extracted from infected Vero E6 SARS-CoV-2 culture by RIPA buffer (ThermoFisher Scientific, US) and PMSF extraction (Sigma-Aldrich, US). The sample was centrifuged at 3000×g at 4°C for one hour, and the samples were subjected to polyacrylamide gel electrophoresis (PAGE).

Samples were diluted and mixed with a Laemmli loading solution (Bio-Rad Laboratories Inc, US) and were denatured at 100°C for five minutes before loading on the polyacrylamide gel. Viral proteins were separated on 12% gradient polyacrylamide gels. The transfer of proteins to the PVDF membrane (Sigma-Aldrich, US) was performed with a Trans-Blot Turbo system (Bio-Rad Laboratories Inc, US) for seven minutes. ^36^

Western blot was performed by using antibody serum from an immunized rabbit with visualization and with HRP-conjugated anti-rabbit IgG polyclonal antibody with 1:10.000 concentration (Cat. no. 611-103-122, Rockland, USA) and DAB (Rockland, USA). A comparison of the western blot was made with convalescent serum that was collected from a volunteer COVID-19 convalescent subject with visualization by HRP-conjugated anti-human IgG polyclonal antibody with 1:10.000 concentration (Cat. no. 611-103-123, Rockland, USA) and DAB with 1X concentration (DAB-10, Rockland, USA). This human serum was used to demonstrate human immune response to SARS-CoV-2.

### Ethics statement

Ethical clearance for this study was approved by the Institutional Review Board of the Dr Soetomo General Hospital, Surabaya (IRB Number IRB00008635), Ethical Clearance No. 0099/LOE/301.4.2/VIII/2020. During hospitalization, the subject or subject’s guardian was informed about the study and gave their written informed consent for participating in this study and use for publication.

## Results

In this study, we conducted the characterization of SARS-CoV-2 East Java isolate, Indonesia. ^37^

### Virus isolation and culture

SARS-CoV-2 CPE appeared in the form of rounding, elevation, and detachment of Vero E6 monolayer cell culture as reported previously. ^38^ Virus passage was performed periodically until stable cytopathic effect patterns were established (5–6 days’ interval) (see Figure 1).

### Growth characteristics and viral morphology

Growth characteristics suggested a more adapted virus culture along with the blind passage progression and intervals with shorter CPE formation times. Moreover, TICD50 evaluation of #35 isolate through plaque forming assay and Reed and Muench method showed 10 ^13.76^ TICD50.

### Scanning transmission electron microscopy

**Figure 2.**
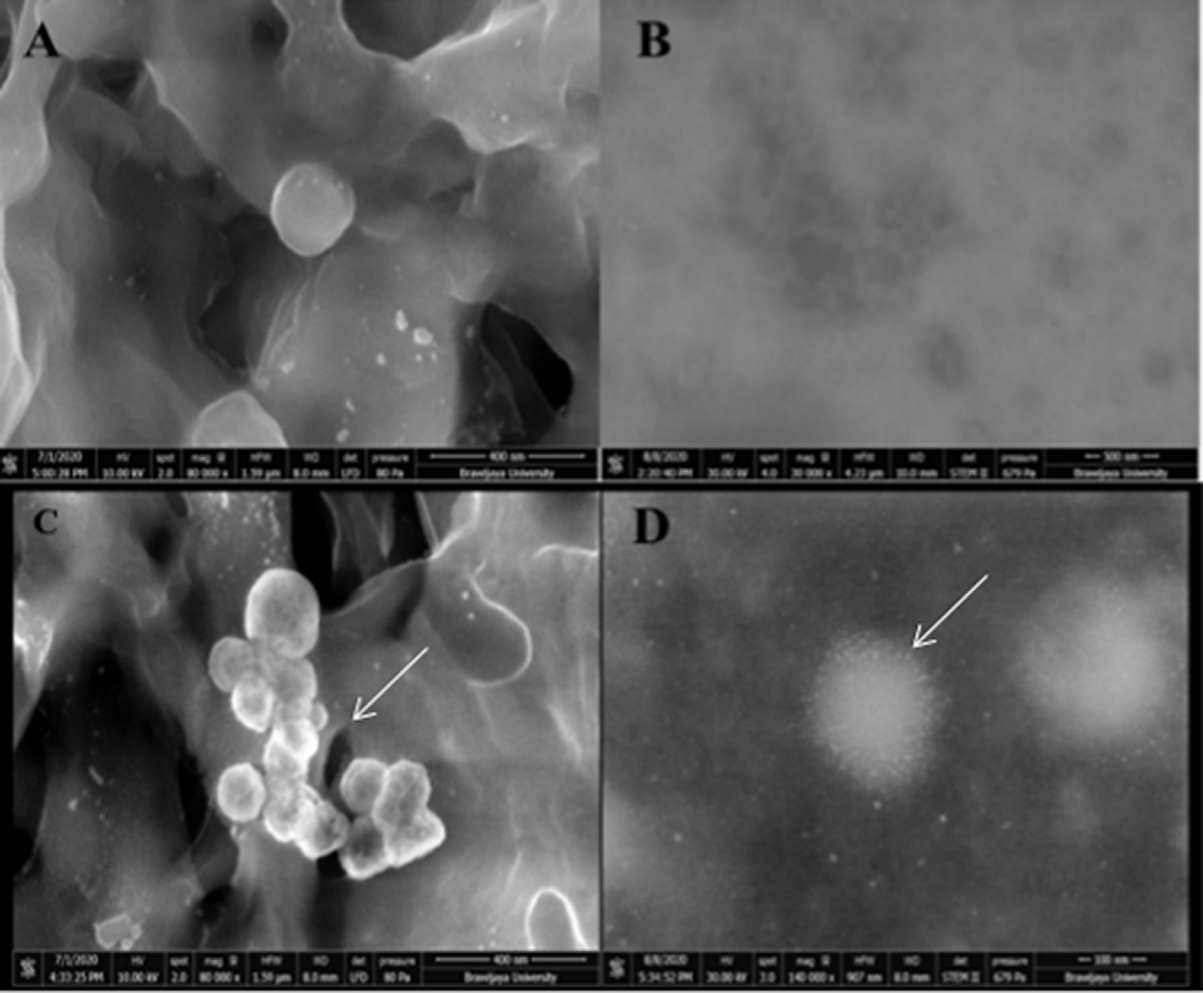
The result of scanning transmission electron microscopy (STEM) (A, B) uninfected cells medium acted as a blank control; (C, D) the morphology of severe acute respiratory syndrome coronavirus 2 (SARS-CoV-2) infected patients under scanning electron microscopy (SEM) 80,000× and scanning transmission electron microscopy (STEM) (FEI Quanta 650 FEG (FEI, USA).

FEI Quanta 650 FEG electron microscopy was performed in low vacuum SEM mode (80 Pa; 10 kV) at 80,000× magnification, and MAPS (Modular Automated Processing System) software observation was able to find several ovoid-shaped multilobulated viral particles that were observed in SEM, with an estimated diameter ranging from 125.8-199.1 nm between filter membrane fibers. Further analysis in STEM showed SARS-CoV-2 viral particles with its surface spike proteins and internal diameter ranging from 120–200 nm (see Figure 2).

### Reverse transcription-polymerase chain reaction (RT-PCR)

**Figure 3.**
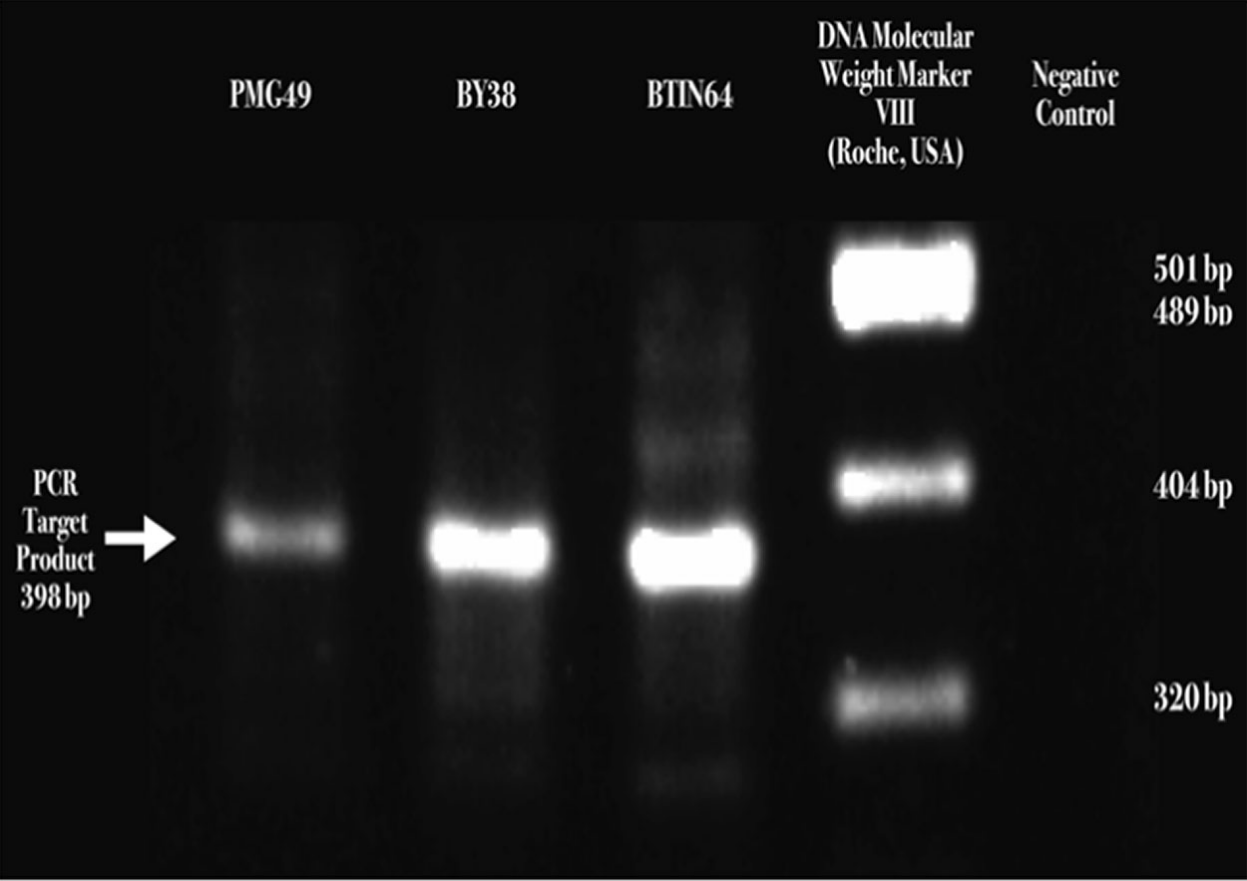
(A) Severe acute respiratory syndrome coronavirus 2 (SARS-CoV-2) S1/S2 cleavage site Receptor Binding Domain (RBD) detected by two-step reverse transcription-polymerase chain reaction (RT-PCR), DNA electrophoresis ethidium bromide staining, and visualized through the Gel Doc XR+ gel documentation system (Bio-Rad, USA).

The visualization of a 398-base pair size band fragment of Receptor Binding Domain (RBD) sequences confirmed SARS-CoV-2 presence. (Roche, USA) (see Figure 3 and Raw RT-PCR Results of SARS-COV-2 ^39^).

### Spike gene analysis

Spike gene analysis of three isolates from three patients found that only the D614G mutation was detected among the isolates, although in #35 isolates D215Y and E484D mutations were also present. Based on whole genome analysis, those three isolates were included in clade 20A, and two isolates were included in lineage B.1.6, with one isolate belonging to lineage B.1.4.7 ( Table 1).

**Table 1.** Isolate’s lineage, clade, passage, amino acid mutation, reference sequence, and accession number.

No	Isolates	Lineage	Clade	Passage	Amino Acid Mutation	Wuhan (Refseq)	GISAID Accession ID	NCBI Accession ID
1	35-A	B.1.6	20A	2	N: D128Y ORF1b: P314L, V345L, N1047D ORF3a: Q57H ORF8: G66X, K68X S: D215Y, E484D, D614G	N: D128D ORF1b: P314P, V345V, N1047N ORF3a: Q57Q ORF8: G66G, K68K S: D215D, E484E, D614D	EPI_ISL_1364466	MZ026853
2	BY38	B.1.4.7	20A	15	ORF1b: P218L, P314L ORF3a: Q57H S: D614G	ORF1b: P218P, P314P ORF3a: Q57Q S: D614D	EPI_ISL_1366083	MZ026854
3	BTIN64	B.1.6	20A	15	ORF1b: P314L S: D614G	ORF1b: P314P S: D614D	EPI_ISL_1366238	MZ026855

### Isolation and characterization of protein immunogenic of SARS-CoV-2

**Figure 4.**
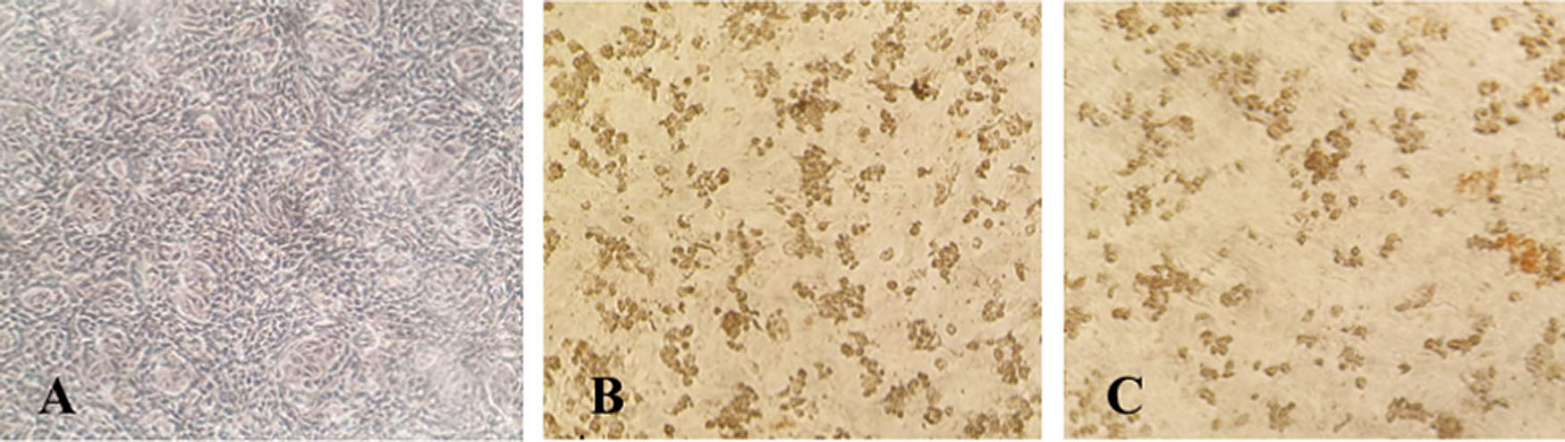
Severe acute respiratory syndrome coronavirus 2 (SARS-CoV-2) Vero E6 infected cells, immunocytochemistry using (A) uninfected Vero E6 cells as control; (B) rabbit antibody serum, (C) human antibody serum (100× magnification).

Immunocytochemistry staining suggested that antibody serum from immunized rabbit and human natural infection serum could detect SARS-CoV-2 infected cells in virus culture, indicated by brown stained cells that were observed in light-inverted microscopy with 100x magnification (Nikon TMS, Tokyo, Japan) (see Figure 4).

**Figure 5.**
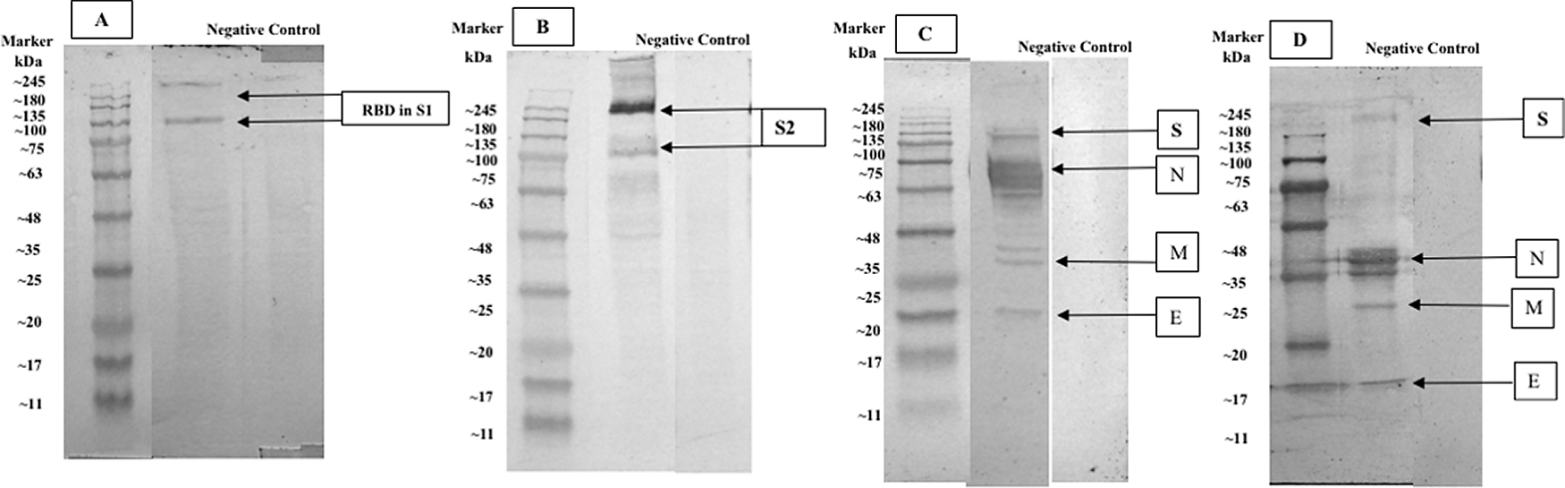
Severe acute respiratory syndrome coronavirus 2 (SARS-CoV-2) western blot staining with anti-SARS-CoV-2 Receptor Binding Domain (RBD) monoclonal antibody for detecting S1 protein (MAB10540, RnD System) (8% gel); (A): western blot staining with anti-SARS-CoV-2 S2 monoclonal antibody (MAB10557, RnD System) (8% gel); (B): western blot staining with rabbit antibody serum (12% gel); (C): western blot staining with human convalescent serum from the COVID-19 infection (8% gel); (D): (S) labeled for spike protein, (N) labeled for nucleoprotein protein, (M) labeled for membrane protein, (E) labeled for envelope protein.

Comparison of western blot staining with antibody serum from immunized rabbit and human natural infection serum that was collected from a volunteer COVID-19 convalescent subject showed several main immunogenic proteins that were detected, including spike glycoprotein (S), nucleocapsid protein (N), membrane glycoprotein (M), accessory 3a protein, and envelope protein (E). Both western blot comparisons between rabbit antibody serum and human convalescent serum from the second wave of COVID-19 in Surabaya showed a relatively similar protein immunogenic capacity (see Figure 5). ^37, 40^

## Discussion

In this study, SARS-CoV-2 isolation was achieved by inoculation in Vero E6 cells and serial blind passages. Observation of phase contrast microscopy suggested CPE with a characteristic of rounding, syncytium formation, and cell detachment with plaque formation. ^26^ Evaluation during virus passage exhibited a shorter time for developing CPE in line with increasing passage numbers that indicated a more adapted virus culture. The serial passage of virus in the culture exhibit with shorter CPE formation indicated increases in virus titers, adaptation, and more virus replication efficiency. ^41^

SARS-CoV-2 infection was confirmed by SEM, STEM, and RT-PCR examination. SEM observation showed a three-dimensional structure of viral particles without spike protein visualization with a viral particle diameter that was in line with previously reported cases. STEM observation showed cross-sectional images of viral particle structure, with spike protein visualization with a viral particle diameter relatively similar to a previous study that reported SARS-CoV-2 morphology using transmission electron microscope (TEM). ^26^ However, interestingly, in this study, we found a rounded multilobulated SARS-CoV-2 viral particle structure (pleomorphic). SEM could detect a viral particle structure that was trapped in the filter fibers. However, the visualization was limited in the three-dimensional structure and could be biased. Fortunately, with STEM, the protein structure of the SARS-CoV-2 spike protein could be visualized more clearly than with SEM. In addition, adequate viral particle visualization enables viral particle internal diameter measurement. The confirmation of SARS-CoV-2 was done by a RT-PCR that detected the SARS-CoV-2 RBD that is located in the S1 subunit of the SARS-CoV-2 spike protein. ^23^ Thus, the analysis of the SARS-CoV-2 spike gene shows the mutation of D614G that increases SARS-CoV-2 infectivity, whereas the E484D mutation was correlated with human immune serum neutralization resistance. ^21^ Whole genome sequence analysis suggested that three isolates were included in clade 20A, and two isolates were included in lineage B.1.6, with one isolate belonging to lineage B.1.4.7. Lineage B.1 is the most abundant lineage that circulates in the environment. Evaluation of genetic stability could be evaluated during virus passage in the *in vitro* setting, as it is reported that genetic mutation may also occur besides natural infections and transmission. ^42^

SARS-CoV-2 infected Vero E6 cells showed a brown color as a positive marker, detected by means of immunized rabbit serum antibody and human natural infection serum antibody immunocytochemistry. Based on this result, evaluation of virus infectivity could be done. ^3^ The comparison between the western blot analysis with immunized rabbit serum antibody and human natural infection serum may suggest that immune response towards SARS-CoV-2 infection tends to be relatively similar. Therefore, these results imply that SARS-CoV-2 immunogenic proteins may be suitable with a natural infection immune response.

Western blot examination suggested that the main protein of SARS-CoV-2, which consisted of S, N, M, accessory 3a, and E proteins, showed that these proteins possessed immunogenic capability. ^3^ Yet, the main protein that plays an important role in SARS-CoV-2 infection, especially in the binding-to-host cells through ACE2 receptors, is spike protein. ^4^ The binding of antibodies to this SARS-CoV-2 main protein could mediate a SARS-CoV-2 immune response and possibly provide immune protection towards COVID-19.

## Conclusion

Based on molecular characterization and immunogenicity of SARS-CoV-2 East Java, Indonesia showed high titer and it has mutation in some regions. Further study is still required to examine the genetic and immunogenic stability for seed vaccine exploration and it requires more investigation and clinical validation.

## Data availability

### Underlying data

Figshare: Underlying data for ‘Characterization of SARS-CoV-2 East Java isolate, Indonesia’, https://doi.org/10.6084/m9.figshare.14703567.v1. ^37^

This project contains the following underlying data:

‘Severe acute respiratory syndrome coronavirus 2 (SARS-CoV-2) western blot staining with anti-severe acute respiratory syndrome coronavirus 2 (SARS-CoV-2) Receptor Binding Domain (RBD) monoclonal antibody for detecting S1 protein’

• Figure 5A. tiff
• Figure 5B. tiff
• Figure 5C. tiff
• Figure 5D. tiff

Figshare: https://doi.org/10.6084/m9.figshare.14703579.v1. ^39^

This project contains the following underlying data:

‘Raw RT-PCR Results of SARS-COV-2’

• Raw PCR.tiff

Figshare: https://doi.org/10.6084/m9.figshare.14703582.v1. ^40^

This project contains the following underlying data:

‘Gels for the Western blot (Raw) Severe acute respiratory syndrome coronavirus 2 (SARS-CoV-2) western blot staining with anti-Severe acute respiratory syndrome coronavirus 2 (SARS-CoV-2) Receptor Binding Domain (RBD) monoclonal antibody for detecting S1 protein’

• Raw WB A.tiff
• Raw WB B.tiff
• Raw WB C.tiff
• Raw WB D.tiff

Data are available under the terms under the terms of the Creative Commons Attribution 4.0 International license (CC-BY 4.0).

## Consent

Written informed consent for publication of the patients’ details was obtained from the patients.
