# Supplementary material for: Characterization of SARS-CoV-2 East Java isolate, Indonesia
Source: F1000Res. 2021 Jun 16;10:480. [Version 1] doi: 10.12688/f1000research.53137.1 (PMC8453313; doi:10.12688/f1000research.53137.1)
Supplement: Peer review history

# Peer review: Reviewer response for version 1

**DOI:** 10.5256/f1000research.56490.r93419

**Published:** 2021-09-29

**Authors:** Marina Derkho

**Affiliations:** 1 South Ural State Agrarian University, Troitsk, Russian Federation

1. The topic of the article is very relevant at the present time. Therefore, when describing the methods, it is desirable to give a scheme of research design.
2. Statistical analysis may not be available in this article.
3. In Figure 4, you can more accurately indicate the presence of detected changes.
4. The conclusions do not reflect the presented data

Is the work clearly and accurately presented and does it cite the current literature?

Yes

If applicable, is the statistical analysis and its interpretation appropriate?

Not applicable

Are all the source data underlying the results available to ensure full reproducibility?

Partly

Is the study design appropriate and is the work technically sound?

Yes

Are the conclusions drawn adequately supported by the results?

No

Are sufficient details of methods and analysis provided to allow replication by others?

Partly

Reviewer Expertise:

NA

I confirm that I have read this submission and believe that I have an appropriate level of expertise to confirm that it is of an acceptable scientific standard, however I have significant reservations, as outlined above.

# Peer review: Reviewer response for version 1

**DOI:** 10.5256/f1000research.56490.r93422

**Published:** 2021-09-20

**Authors:** Lidia Audrey Rocha Valadas

**Affiliations:** 1 University of Buenos Aires, Buenos Aires, Argentina

This is a interesting study that investigates and characterizes SARS-CoV-2 isolated from East Java, Indonesia. In general, the manuscript is well designed and and can be reproducible for others. The methods and results, specially, were very well conducted.

 Some of my suggestions below:

- I suggest a little change on the title, for example: Characterization of SARS-CoV-2 isolated in East Java-Indonesia.
- I suggest a flowchart  on the Methods-Subject characteristics.
- It's missing the ethical aspects (approval number of the project). It's cited just the consent signed.
- Please provide the limitations.

Is the work clearly and accurately presented and does it cite the current literature?

Yes

If applicable, is the statistical analysis and its interpretation appropriate?

Not applicable

Are all the source data underlying the results available to ensure full reproducibility?

Yes

Is the study design appropriate and is the work technically sound?

Yes

Are the conclusions drawn adequately supported by the results?

Yes

Are sufficient details of methods and analysis provided to allow replication by others?

Yes

Reviewer Expertise:

Dentistry, Saliva, Biofilms

I confirm that I have read this submission and believe that I have an appropriate level of expertise to confirm that it is of an acceptable scientific standard.

# Peer review: Reviewer response for version 1

**DOI:** 10.5256/f1000research.56490.r87787

**Published:** 2021-09-16

**Authors:** Aulanni’am Aulanni'am

**Affiliations:** 1 Department of Biochemistry, Faculty of Mathematics and Natural Sciences, Universitas Brawijaya, Malang, Indonesia

This study showed the important result that can be studied by others. SARS-Cov2 isolate plays important roles for disease development so it will help for progressive effect prevention that lead to mortality. The technique applied in this work also reliable to follow in standard infectious laboratory. By identification of local isolates, it would be useful to identify disease transmission as well as the impact to disease progressive. They used standard method that can be followed by other researchers with the same objective

Is the work clearly and accurately presented and does it cite the current literature?

Yes

If applicable, is the statistical analysis and its interpretation appropriate?

Partly

Are all the source data underlying the results available to ensure full reproducibility?

No source data required

Is the study design appropriate and is the work technically sound?

Yes

Are the conclusions drawn adequately supported by the results?

Yes

Are sufficient details of methods and analysis provided to allow replication by others?

Yes

Reviewer Expertise:

Biochemistry, Molecular diagnostic, medical devices development, Biomedics

I confirm that I have read this submission and believe that I have an appropriate level of expertise to confirm that it is of an acceptable scientific standard.
